# The Evolutionary Relationship between Microbial Rhodopsins and Metazoan Rhodopsins

**DOI:** 10.1155/2013/435651

**Published:** 2013-02-11

**Authors:** Libing Shen, Chao Chen, Hongxiang Zheng, Li Jin

**Affiliations:** State Key Laboratory of Genetic Engineering and Key Laboratory of Contemporary Anthropology of Ministry of Education, School of Life Sciences, Fudan University, Shanghai 200433, China

## Abstract

Rhodopsins are photoreceptive proteins with seven-transmembrane alpha-helices and a covalently bound retinal. Based on their protein sequences, rhodopsins can be classified into microbial rhodopsins and metazoan rhodopsins. Because there is no clearly detectable sequence identity between these two groups, their evolutionary relationship was difficult to decide. Through ancestral state inference, we found that microbial rhodopsins and metazoan rhodopsins are divergently related in their seven-transmembrane domains. Our result proposes that they are homologous proteins and metazoan rhodopsins originated from microbial rhodopsins. Structure alignment shows that microbial rhodopsins and metazoan rhodopsins share a remarkable structural homology while the position of retinal-binding lysine is different between them. It suggests that the function of photoreception was once lost during the evolution of rhodopsin genes. This result explains why there is no clearly detectable sequence similarity between the two rhodopsin groups: after losing the photoreception function, rhodopsin gene was freed from the functional constraint and the process of divergence could quickly change its original sequence beyond recognition.

## 1. Introduction

Rhodopsin is a class of proteins whose common features are a seven-transmembrane alpha-helix apoprotein and a cofactor of retinal [1, 2]. Retinal works as a rhodopsin's chromophore which is responsible for light absorption. It reversibly and covalently binds to a lysine in the seventh helix of apoprotein. So to speak, the protein part of rhodopsin is its structural foundation while the retinal is rhodopsin's functional backbone. Rhodopsins are ubiquitously found in three domains of life—archaea, eubacteria, and eukaryotes [3–7]. According to their protein sequences, rhodopsins can be classified into two groups—Type 1 rhodopsins and Type 2 rhodopsins [2]. Type 1 rhodopsins exist in single-celled organisms while Type 2 rhodopsins only appear in multicellular animals. For convenience, we call Type 1 rhodopsins microbial rhodopsins and Type 2 rhodopsins metazoan rhodopsins in this study. Microbial rhodopsins function as phototaxis receptors (sensory rhodopsin), light-driven proton or chloride ion transporters (bacteriorhodopsin and halorhodopsin) [2, 3, 5, 6, 8]. Metazoan rhodopsins mainly function as visual receptors in animal's eyes such as rod or cone opsins [9–11]. Like microbial rhodopsins, metazoan rhodopsins also perform nonsensory functions. Melanopsin, expressed in brain and eyes, may be involved in circadian rhythms and papillary reflex [12]. Neuropsin (Opn5) is expressed in predominantly neural tissues [13]. Encephalopsin is expressed in brain and visceral organs [14]. RGR opsin, expressed in the retinal pigment epithelium (RPE) and Müller cells, functions as the photoisomerase [15, 16]. Peropsin is expressed in the retinal pigment epithelium (RPE) cells [17]. So far researchers have identified nine subgroups of nonvisual opsins in Metazoa [18–21].

The evolutionary relationship between microbial rhodopsins and metazoan rhodopsins is difficult to decide, because they show no clearly detectable identity at sequence level. Although lacking in sequence identity cannot be used to prove that they are not homologous proteins, sequence identity is the cornerstone for conventional knowledge of protein homology [22]. Due to evolutionary divergence, the sequence identity in different homologous proteins decreases with time. Our ability to detect sequence homology in related proteins depends on their divergence rate and evolutionary distance [23]. Using PAM matrix, Dayhoff et al. show that the limitation of sequence identity for deducing protein homology is around 20% identity [23]. If two proteins share less than 20% sequence identity, it means either they are not homologous proteins or their common origin is obliterated in evolution. 

There are two possible evolutionary scenarios for microbial rhodopsins and metazoan rhodopsins: (1) using retinal as chromophore, binding retinal with a lysine and similar seven-transmembrane domain are the result of convergent evolution; (2) their common features are the legacy of a common ancestor, yet their sequence identity is hardly detectable because of the quick and/or longtime divergence.

To investigate the evolutionary relationship between microbial rhodopsins and metazoan rhodopsins, we have to bypass the problem of lacking sequence similarity. Fitch developed a statistical method to distinguish homologous proteins from nonhomologous ones [24]. His method compares the ancestral state from one protein group with the ancestral state from another. It circumvents the need of sequence identity to decide the evolutionary relationship between two groups of proteins. In this study, we used his method to test whether microbial rhodopsins and metazoan rhodopsins are homologous proteins or not.

## 2. Materials and Methods

### 2.1. Structure Data

A direct search in PDB database came back with two metazoan rhodospins and five microbial rhodopsins with structure data (Table 1).

### 2.2. Sequence Data

The whole genome protein sequences and corresponding cDNA sequences for twenty-seven metazoan species were downloaded from Ensembl database, NCBI database, and VectorBase [25]. These species cover seven phyla—Porifera, Cnidaria, Nematoda, Arthropoda, Chordata, Hemichordata, and Echinodermata. The species in Chordata also represented major classes in this phylum. We used a Perl script to extract the longest transcripts for each genome in this study.

### 2.3. BLAST and FASTA Search for Rhodopsin Genes in Genome Data

We used BLAST to search for rhodopsin genes in microbial genomes [26]. Using five microbial rhodopsins with structure data as queries, we searched the complete microbe genome database, fungi genome database, and green algae genome database on NCBI website. The BLAST parameters were set as follows: max target sequences were 500, expect threshold was 0.001, and the others were default.

We used FASTA 3.5 to search for rhodopsin genes in each metazoan genome [27]. Two metazoan rhodopsins with structure data served as queries. The E-value for FASTA search was set as 0.001.

Hits in BLAST or FASTA search result were aligned back to query sequences using MUSCLE with default parameters [28]. The hits were identified as candidate rhodopsins only when they share a conserved retinal-binding lysine in the seventh helix as the same position as queries. We removed redundant candidate hits and any sequence shorter than 200 amino acids or longer than 1000 amino acids.

### 2.4. Structure Alignment

Using their PBD files, two metazoan rhodospin protein structures and five microbial rhodopsin protein structures were aligned with CE-MC multiple protein structure alignment server with default parameters [29]. 

### 2.5. Sequence Alignment

Microbial or metazoan rhodopsin protein sequences were aligned using MUSCLE with default parameters [28]. All nucleotide sequences in this study were aligned according to their protein sequence alignment result.

### 2.6. Test Region Selection

Although there is no clearly detectable sequence identity, protein structure is something comparable between microbial and metazoan rhodopsins. The selection of test region between microbial and metazoan rhodopsins was based on their structure alignment. The problem we encountered here is that structure data are far scarcer than sequence data in both groups of rhodopsins. Only two metazoan rhodospins and five microbial rhodopsins have structure data. So we have to use their structure alignment as a guide to infer seven-transmembrane domain in their sequence alignment.

All microbial rhodopsins share a clearly detectable sequence homology as well as all metazoan rhodopsins, so sequence alignment result is reliable within microbial or metazoan group. However, structure alignment result does not always coincide with sequence alignment result; that is, the positional homology proposed by microbial structure alignment may not be the same one proposed by microbial sequence alignment. Our solution is that we first aligned all microbial rhodospin sequences using MUSCLE. Then we picked out five microbial rhodopsin sequences with structure data in MUSCLE alignment result and compared their sequence alignment with their structure alignment. By doing so, we could identify the positional homology agreed by both alignment methods. We repeated this practice in metazoan rhodopsins using squid and bovine rhodopsins' structure alignment as a guide. The final test region is the alignment result agreed by both structure and sequence alignments.

### 2.7. Phylogenetic Analysis and Ancestral State Inference

Neighbor-joining, Bayesian, and maximum-likelihood methods were used to construct phylogenetic tree for microbial or metazoan rhodopsins. ProtTest was used to select evolution models for our phylogenetic analyses [30]. MEGA 5 was used to construct NJ tree with “pairwise deletion” option and “JTT” model [31]. Rates and patterns were set as “Gamma Distributed”, and Gamma parameter was set as “4”. Bootstrap method was used to test phylogeny, and number of bootstrap replications was set as “500”. PhyML 3.0 was used to construct ML tree with “WAG” model [32]. Proportion of invariable sites and gamma shape parameter were estimated from alignment result. Approximate likelihood-ratio test was used to test for branch reliability [33]. MrBayes 3.1.1 was used to construct Bayesian tree with “WAG” model [34]. We ran for 500,000 generations and sampled posterior probability trees every 1000 generations. We summarized 25% of both parameter values and trees to get the consensus tree.

PHYLIP package was used to construct Fitch-Margoliash tree for rhodopsin genes within each metazoan species [35]. Within-species rhodopsin tree was built with “JTT” model and tested with 100 bootstrap replicates. 

Phylogenetic trees served as the evolutionary history for our ancestral state inference. Parsimony method was used to infer ancestral states [24]. We wrote a Perl script to implement this method.

### 2.8. Test for Relatedness in Ancestral States

The test for relatedness in two ancestral states is a statistic method Fitch devised in his 1970 paper [24]. The basic idea behind this test is that the probability of relatedness can be calculated by comparing the observed mutation distance between two ancestral states with the expected mutation distance between them. The observed mutation distance is the actual nucleotide differences between two ancestral states. The expected mutation distance between two ancestral states is the probability of randomly chosen disjoint nucleotide sets between them multiplied by the length of their sequence. The standard deviation between two distances is the square root of expected distance multiplied by the probability of randomly chosen intersectant nucleotide sets between them. The number of standard deviations between the observed mutation distance and the expected mutation distance follows normal distribution. The probability of its value could be found in the table of normal probability and it is used as the probability of significance.

## 3. Results

### 3.1. Structural Homology between Microbial Rhodopsin and Metazoan Rhodopsin

**Figure 1:**
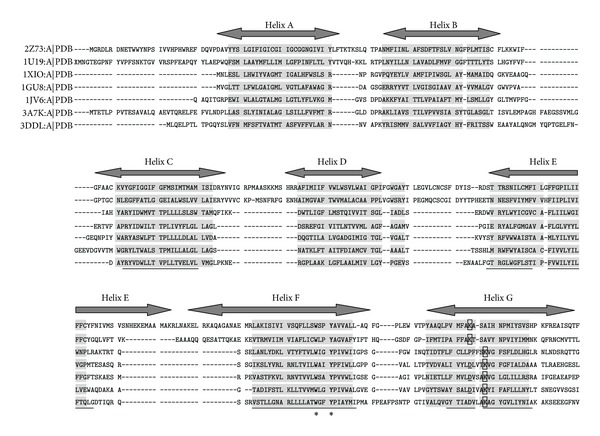
Structure alignment of squid rhodopsin (2Z73:A*|*PDB, metazoan rhodopsin), bovine rhodopsin (1U19:A*|*PDB, metazoan rhodopsin), *Anabaena* sensory rhodopsin (1XIO:A*|*PDB, microbial rhodopsin), *Natronomonas* sensory rhodopsin II (1GU8:A*|*PDB, microbial rhodopsin), *Halobacterium salinarum* bacteriorhodopsin (1JV6:A*|*PDB, microbial rhodopsin), *Natronomonas* halorhodopsin (3A7 K:A*|*PDB, microbial rhodopsin), and *Salinibacter ruber* xanthorhodopsin (3DDL:A*|*PDB, microbial rhodopsin). Squid rhodopsin is used as the template for delineating seven-transmembrane helices. Shaded residues are structural homologues. Conserved tryptophan and tyrosine in WXXY motif are marked with black asterisks. The retinal-binding lysine is in bold style and boxed. The aspartic acid in microbial rhodopsin corresponding to the retinal-binding lysine in metazoan rhodopsin is in bold style and underlined. The test region is marked with thin lines.

The structure alignment of five microbial rhodopsins and two metazoan rhodospins shows that all rhodopsins share a remarkable structural homology (Figure 1). Seven-transmembrane helices are conserved within microbial or metazoan rhodopsins and between them. Although there is no clearly detectable sequence homology between these two groups of rhodopsins, the structure alignment reveals that they share a conserved WXXY sequence motif in the sixth helix. Interestingly, the lysine that binds retinal in the seventh is not structurally conserved and locates in different position between them. There is also an/a insertion/deletion in the seventh helix between these two groups of rhodopsins, which is just one amino acid before the crucial lysine in microbial rhodopsins (insertion) or one amino acid after the crucial lysine in metazoan rhodopsins (deletion). So the position of retinal-binding lysine shifts three amino acids forward in metazoan rhodopsins.

### 3.2. Rhodopsin Genes in Microbial and Metazoan Genomes

BLAST search for microbial rhodopsins came back with 62 microbial rhodopsins (See Table S1 in Supplementary Material available online at http://dx.doi.org/10.1155/2013/435651). FASTA search for metazoan rhodopsins came back with 227 metazoan rhodopsins from 25 species (Table 2).

In 62 microbial rhodopsins, thirty-five of them are from bacteria, twenty-four are from archaea, and three are from eukaryotes. Bacterium *Salinibacter ruber M8* and archaea *Haloarcula marismortui ATCC 43049* have four different copies of rhodopsin gene. One bacterium species and four archaea species have three different rhodopsin genes. Eleven microbial species have two different rhodopsin genes. Among three eukaryotic microbial rhodopsins, two of them are from single-celled green alga *Chlamydomonas reinhardtii* and one is from encapsulated yeast *Cryptococcus neoformans* var. *neoformans*.

Table 2 shows the number of rhodopsin genes in each metazoan species. We named rhodospin genes in numeric order within each metazoan species. The number of rhodopsin genes varies drastically in each metazoan species. In insects, malaria mosquito has nine rhodopsin genes while body blouse only has three. There is no rhodopsin gene found in sponge* Amphimedon queenslandica* and nematode *Caenorhabditis elegans*, although they do have rhodopsin-related genes.

### 3.3. Final Test Region

The final test region we selected is the consistent alignment result between structure and sequence alignments. There is no consistent region found in helices A, B, or D. In helix C, there is an 18-amino acid consistent region. In helix E, there are two consistent regions: one is 11 amino acid long and the other is 14 amino acid long. In helix F, there is a 25-amino acid consistent region. In helix G, there is an 18-amino acid consistent region. The total test region is 86 amino acid long and equals 258 nucleotides. 

### 3.4. The Evolutionary History and Ancestral State Inference in Metazoan Rhodopsins

 We used three different methods to construct phylogenetic trees for all metazoan rhodopsins in this study. *Hydra* rhodopsins serve as an outgroup to root metazoan trees. In our study, *Hydra* is the only animal from Cnidaria. It is the basal phylum to Arthropoda, Chordata, Hemichordata, and Echinodermata. Rooted with *Hydra* rhodopsins, three trees show three different overall topologies. Neighbor-joining tree shows all rhodopsin genes divided into three major clades except *Hydra* rhodopsins (Supplemental Figure 1). One clade mainly consists of chordate rhodopsins and no arthropod rhodopsins. The other two clades contain both chordate and arthropod rhodopsins. Maximum-likelihood tree shows a different evolutionary history from NJ tree (Supplemental Figure 2). ML tree has four major clades instead of three. Bayesian tree shows a more complicated evolutionary history (Supplemental Figure 3). Three separate clades in NJ tree are mixed in Bayesian tree. We did not know which tree is the most reliable one in all three trees. Three trees produced three different ancestral states. Only one state is true, because all metazoan rhodopsins share only one evolutionary history.

**Figure 2:**
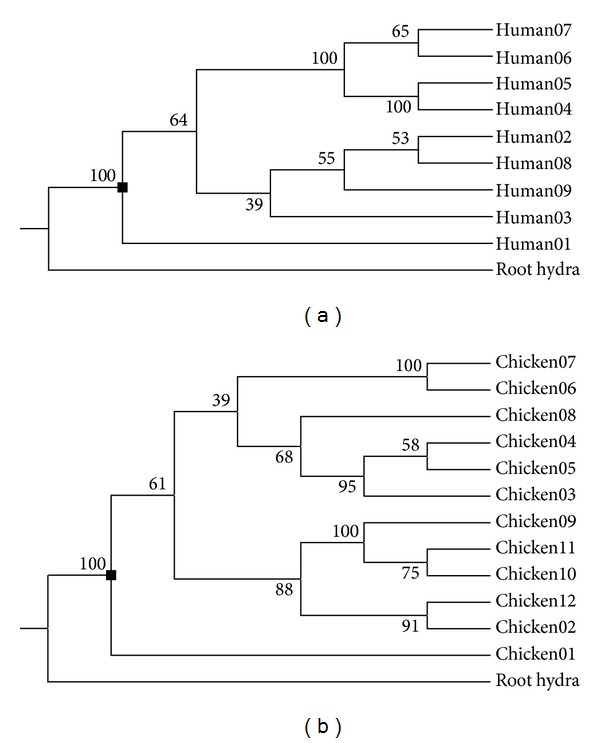
(a) Fitch-Margoliash tree for all rhodopsin genes in human. (b) Fitch- Margoliash tree for all rhodopsin genes in chicken. *Hydra* rhodopsin gene serves as outgroup. The numbers adjacent to tree nodes are bootstrap values. The tree node where ancestral state is built on is marked with a filled black square ■.

 In order to get reliable ancestral state, we constructed the phylogenetic tree for rhodopsins within each metazoan species instead of for all metazoan rhodopsins (Figures 2(a) and 2(b)). By reducing the number of taxa in tree construction, we could get more reliable trees for ancestral state inference. Nevertheless, by doing so, we had to infer one ancestral state for each metazoan species. Using one *Hydra* rhodopsin as an outgroup, we constructed 24 metazoan rhodopsin trees and inferred 24 ancestral states based on these trees. Eighteen of them are possible metazoan rhodopsin's ancestral states in Chordata. Four of them are possible ancestral states in Arthropoda. Two of them are possible ancestral states in Hemichordata and Echinodermata.

### 3.5. The Evolutionary History and Ancestral State Inference in Microbial Rhodopsins

We also used three different methods to construct phylogenetic trees for all microbial rhodopsins. Three microbial trees are consistent in overall topologies, although they differ in the position of one branch which contains six bacteria rhodopsins (Supplemental Figures  4, 5, and 6). The problem is that bacteria and archaea are sister clades in biological systematics. It means that we are unable to root microbial trees. If we could not decide an outgroup for microbial trees, we would not be capable of inferring any ancestral state with them.

**Figure 3:**
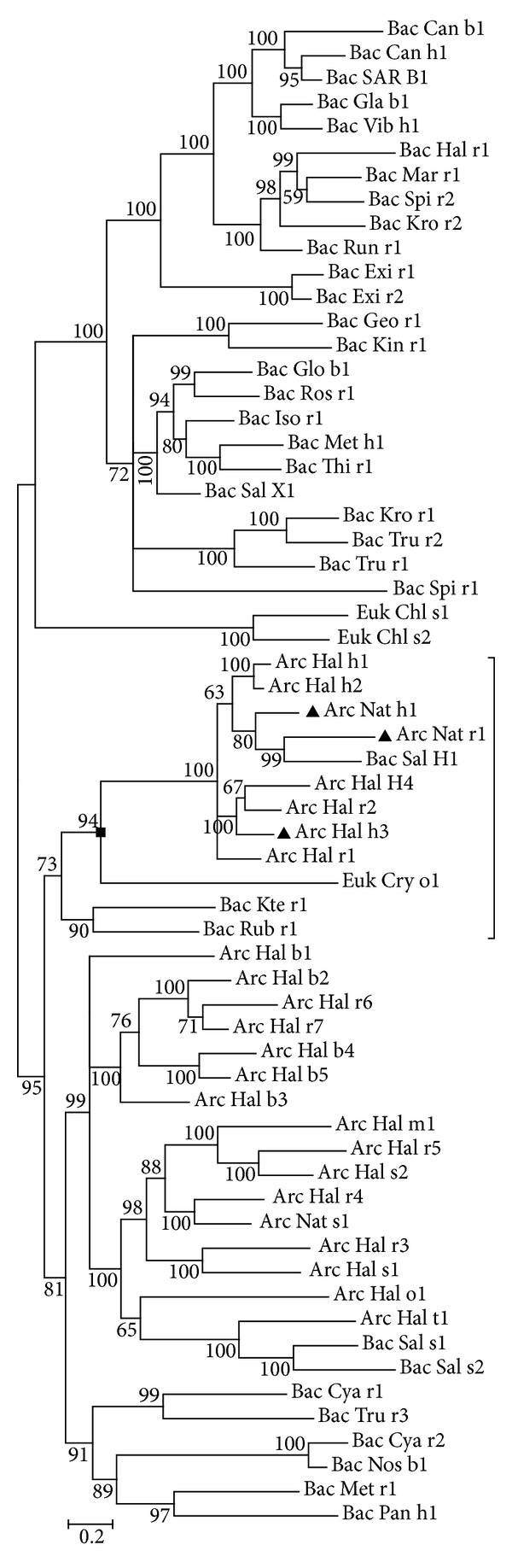
Unrooted Bayesian tree for all microbial rhodopsin genes. The numbers adjacent to the nodes are posterior probability values. The length of branch reflects evolutionary divergence. Microbial rhodopsin genes distantly related to metazoan rhodopsin's ancestral states (>95% quantile) are marked with a filled black triangle ▲. The tree node where microbial rhodopsin's ancestral state is built on is marked with a filled black square ■. In all possible ancestral states on that branch (marked with vertical line), this microbial rhodopsin's ancestral state has the smallest mutation distance with metazoan rhodopsin's ancestral states.

To overcome this problem, we first tried to find which microbial subtree is the most possible candidate tree for ancestral state inference. Using Fitch's method, we tested each extant microbial rhodospin gene with 24 metazoan ancestral states. We found that three microbial rhodopsins are distantly related to metazoan ancestral states with statistical significance (Supplemental Table 2). These three rhodopsins are all located in one single subtree which contains 13 microbial rhodopsins (Figure 3). Then we inferred all possible microbial rhodopsin's ancestral states on this subtree.

### 3.6. The Relatedness between Microbial Rhodopsins' Ancestral States and Metazoan Rhodopsins' Ancestral States

We tested 24 metazoan rhodopsin's ancestral states with all possible microbial rhodopsin's ancestral states on the candidate subtree. Among all inferred microbial rhodopsin's ancestral states, one microbial rhodopsin's ancestral state has the smallest mutation distance with metazoan rhodopsin's ancestral states (Figure 3). This microbial ancestral state is reconstructed upon one fungi rhodopsin, one bacteria rhodopsin, and eight archaea rhodopsins. Test result shows that 13 metazoan rhodopsin's ancestral states are divergently related to it with statistical significance (Table 3). These ancestral states cover Arthropoda, Chordata, Hemichordata, Echinodermata, and two subphyla in Chordata—Tunicata (sea squirt) and Cephalochordata (amphioxus).

## 4. Discussion

### 4.1. Structural Homology versus Common Origin

Microbial rhodopsins and metazoan rhodopsins share a remarkable structural homology in their seven helices (Figure 1). However, the structural homology does not necessarily indicate the common origin. The empirical view of common origin is based on sequence homology. Convergent evolution is also a probable cause for structural homology [36]. Through both structure alignment and sequence alignment, we found that the vast majority of microbial and metazoan rhodopsins share a conserved WXXY sequence motif in the sixth helix. The tryptophan and tyrosine in this motif are crucial amino acids which form retinal-binding pocket in both groups of rhodopsins [37–41]. The conservation of WXXY motif in both groups of rhodopsin can be explained by either convergent evolution or common origin. In this case, common origin seems to be more plausible than convergent evolution. According to PAM matrix, tryptophan is the least mutable amino acid and tyrosine is the fifth-least mutable amino acid [23].

### 4.2. The Convoluted Evolutionary History of Metazoan Rhodopsins

There is only one rhodopsin gene found in acorn worm while there are 35 rhodopsin genes found in zebra fish. No rhodopsin gene found in sponge and nematode indicates that rhodopsin is not essential for the survival of metazoa. However, photoreception capability does grant animals a great advantage for their survival. Nonessentiality and advantage for survival render the evolution of metazoans rhodopsins a birth-and-death process, in which gene duplication event creates new genes and some newly-created genes are kept in genome while others vanish from genome by accumulating deleterious mutations [42]. This process led to the various number of rhodopsin genes in different metazoan species; for example, body louse has three different rhodopsin genes while malaria mosquito has nine, and both of them are insects. It also made divergence and subfunctionalization rampant among duplicated rhodopsin genes. There are at least ten different subgroups of metazoan rhodopsins, and only one subgroup directly functions as visual opsins [11, 18–21]. The birth-and-death process produced a very complicated evolutionary history for metazoan rhodopsins. Due to their convoluted evolutionary history and the large number of sequences used in phylogenetic analysis, we could not acquire an accurate phylogenetic tree for all metazoan rhodopsins. So in ancestral state inference, we used each species' rhodopsin genes to perform phylogenetic analysis in order to build a reliable tree within each metazoan species.

### 4.3. Gene Duplication and Horizontal Gene Transfer in Microbial Rhodopsins

Gene duplication and horizontal gene transfer are common in microbial rhodopsins. Two microbial species have four rhodopsin genes, five species have three rhodopsin genes, and eleven species have two rhodopsin genes (Supplemental Table 1). Both of the gene duplication and horizontal gene transfers contribute to multiple rhodopsin copies in these species. For example, bacterium *Salinibacter ruber M8* has four rhodopsin genes. Its two sensory rhodopsins (Bac_Sal_s1 and Bac_Sal_s2) were the result of a gene duplication event, but they are clustered with archaea rhodopsins in microbial tree. It means that *Salinibacter ruber M8 *got its original sensory rhodopsin from archaea through horizontal gene transfer. Horizontal gene transfer makes the origin of microbial rhodopsins untraceable. The fact that all three domains of life have microbial rhodopsins proposes that microbial rhodopsin is a very ancient gene. It could be as old as life itself.

### 4.4. Are Metazoan Rhodopsins and Microbial Rhodopsins Homologous Genes?

The main purpose of this study is to answer the question: are metazoan rhodopsins and microbial rhodopsins homologous genes? Due to the lack of direct evidence—sequence homology, we tried to answer this question by comparing their ancestral states. The complicated evolutionary history of metazoan rhodopsins made a reliable overall phylogenetic tree hardly possible. We circumvented this problem by building the phylogenetic tree for metazoan rhodopsins within each species. Then using these reliable trees, we inferred one ancestral state for each metazoan species. 

In our 24 metazoan rhodopsin's ancestral states, more than half of them are divergently related to the microbial rhodopsin's ancestral state with statistical significance and less than half of them without statistical significance (Table 2). There are two possible explanations for the reason why the other 11 metazoan rhodopsin's ancestral states show no statistical significance: (1) the birth-and-death process eliminated some basal metazoan rhodopsins in these species. Therefore, their phylogenetic trees only allowed us to trace back to a recent ancestral state instead of a much more ancient one; (2) in these species, the existent metazoan rhodopsins diverge from their ancestor so greatly that there is no traceable information left in their sequences. These two explanations are not mutually exclusive.

For thirteen metazoan rhodopsin's ancestral states divergently related to the microbial rhodopsin's ancestral states with statistical significance, does it mean that metazoan rhodopsin and microbial rhodopsin are homologous genes? By the definition of Fitch's test, the answer is yes. The test region we selected is total 86 amino acids. Within test region, the average mutation distance between existent metazoan and microbial rhodopsin is 119 ± 5 mutations in the first and second codon positions. Assuming one mutation in the first or second codon position would change its coding amino acid, each paired codon in the test region averagely shares about 1.38 mutations between two rhodopsin groups. It explains why we cannot find clearly detectable sequence homology between microbial and metazoan rhodopsins. After ancestral state reconstruction, the shortest mutation distance between microbial and metazoan ancestral states was 63 mutations. It is found between chicken and one microbial ancestral state inferred on nine microbial rhodopsins, with a *P* value of 0.0045. There are total 86 amino acids in the test region. If mutations were evenly distributed in each codon, there would be 63 amino acid differences between microbial and metazoan ancestral states. In another words, the sequence identity between microbial and metazoan ancestral states would be 23 amino acids. 23 divided by 86, it is about 26.7% sequence identity.

In pairwise sequence alignment, over 30% sequence identity is the safe standard for homologous proteins. Proteins sharing from 15% to 30% sequence identity are in the twilight zone, which means their homologous status is still in ambiguity [22]. Even when tracing back in time by reconstructing ancestral states, our result shows that only 26.7% sequence identity might exist in four helices between ancestral microbial and metazoan rhodopsins. In conventional viewpoint, such result still cannot prove that metazoan and microbial rhodopsins are homologous proteins. Using *Hydra* rhodopsin as an outgroup, we can only infer metazoan ancestral rhodopsin states as early as in bilaterian ancestors. Fossil records show that the earliest bilaterian animal appeared about 580 million years ago [43]. However, based on the estimation of nuclear genes, early metazoan divergence can be traced back to 830 million years ago [44]. There is no rhodopsin gene found in sponge, and the closest microbe species related to Metazoa in this study is fungus *Cryptococcus neoformans* var. *neoformans*. So we have at least 250-million-year divergence time between microbial and metazoan ancestral states. Such longtime divergence could explain the low sequence identity between microbial and metazoan ancestral states. Certainly, the low sequence identity could also be seemingly explained by convergent evolution, which means rhodopsin gene appeared independently in microbes and Metazoa. But our result shows that ancestral microbial rhodopsins and ancestral metazoan rhodopsins shared about 26.7% sequence identity in four helices. It is implausible to believe that random mutations would create an almost identical structure by generating long strings of amino acids with similar sequences.

### 4.5. The Position of Retinal-Binding Lysine in the Seventh Helix

The structure alignment of microbial and metazoan rhodopsins shows an intriguing phenomenon: although both groups of rhodopsins have a retinal-binding lysine in the seventh helix, the position of this lysine is not structurally conserved between them (Figure 1). Its position shifts three amino acids forward in metazoan rhodopsins. Once again the different position of retinal-binding lysine could be simply explained by convergent evolution. However, most microbial rhodopsins have an aspartic acid in the position where metazoan rhodopsins have a retinal-binding lysine. In microbial rhodopsins, this aspartic acid functions as a part of counterion which balances the positive charge of retinal-binding lysine [45, 46]. Since structure alignment and ancestral state tests suggest that microbial and metazoan rhodopsins are homologous proteins, it means that this negatively charged aspartic acid in microbial rhodopsin mutated to the positively charged retinal-binding lysine in metazoan rhodopsin. The genetic code for aspartic acid is GAC or GAT while the genetic code for lysine is AAG or AAA. These two amino acids share the same adenine at the second codon position. The second codon position tends to have the slowest mutation rate among three codon positions [47]. It is probable that Asp (GAC or GAT coding) first mutated to Asn (AAC or AAT coding) and then Asn mutated to Lys (AAG or AAA coding) during the evolution of rhodopsin gene.

The retinal-binding lysine in the seventh helix is the most crucial amino acid for rhodospin's photoreception function. It binds the chromophore retinal which is responsible for light absorption [1, 2]. If microbial and metazoan rhodopsins are homologous proteins, their retinal-binding lysine at different positions means that the function of photoreception was once lost during the evolution of rhodopsin gene. In metazoan rhodopsin, rescue mutation of this lysine salvaged the function of photoreception in metazoan rhodopsin. The once-lost lysine explains why there is no clearly detectable sequence homology between microbial and metazoan rhodopsins. During the evolution from single-celled organisms to multicellular animals, the rhodopsin gene in early metazoan ancestor lost retinal-binding lysine and therefore lost its function of photoreception. Loss of function freed the rhodopsin gene from functional constraint, and the process of divergence quickly changed its original sequence beyond recognition. Inexplicably in the later metazoan evolution, one of those loss-function rhodopsin genes managed to retrieve a lysine in its seventh helix through random mutation and therefore rescued its function of photoreception.

## 5. Conclusion

Based on our analysis, we propose that microbial and metazoan rhodopsins are homologous proteins and the function of photoreception was once lost during the evolution of rhodopsin gene. This conclusion may be controversial under the conventional view for homologous proteins. Logically, the view that microbial and metazoan rhodopsins are homologous proteins is the most parsimonious one. It does not require another protein to be the precursor of metazoan rhodopsins. Nature just recycled seven-transmembrane-helix protein for photoreception. However, the alternative view that the nearly identical structure between microbial and metazoan rhodopsins is the result of convergent evolution requires random mutations to create seven-transmembrane-helix domain twice through generating long strings of amino acids with similar sequences. Seven-transmembrane-helix domain does perform other functions than photoreception in Metazoa [48]. They form a large protein family of G-protein-coupled receptors which include metazoan rhodopsin and olfactory receptor. Research shows that most of these seven-transmembrane receptors share a common origin [49]. It is natural for someone to wonder what was the origin of all these seven-transmembrane receptors. There is no ancient seven-transmembrane receptor other than microbial rhodopsins which could be as old as life itself. For those who believe that the identical structure between microbial and metazoan rhodopsins is a result of convergent evolution, they will have to answer such two questions: (1) what was the precursor for all seven-transmembrane receptors in Metazoa; (2) if such a precursor existed, how could random mutations shape it into seven-transmembrane helices through generating long strings of amino acids which are also similar to a subset of microbial rhodopsins? On the other hand, our ancestral state inference failed to provide a decisive sequence identity between microbial and metazoan ancestral rhodopsins. The ambiguous sequence identity could be explained by once-relieved functional constraint and the long divergence time between microbes and metazoa. The divergence-time gap might be filled by using rhodopsin-related genes from basal animals for ancestral state inference. The future genome projects for basal animals could hold the ultimate answer to the question of the evolutionary relationship between microbial rhodopsin and metazoan rhodopsin.

## Supplementary Material

Supplemental table 1: Rhodopsin genes identified in microbial genomes.
Supplemental table 2: Microbial rhodopsin gene divergently related to metazoan rhodopsin's ancestral states and how many metazoan rhodopsin's ancestral states it is related to. Here 95% quantile = 15.25.
Supplemental figure 1: Neighbor-joining tree for all metazoan rhodopsin genes. It is based on JTT model and 4 Gamma parameters. The numbers adjacent to tree nodes are bootstrap values. Hydra rhodopsins serve as outgroup. Green, red and blue clades are three major groups in this tree.
Supplemental figure 2: Maximum-likelihood tree for all metazoan rhodopsin genes. It is based on WAG model and 4 Gamma parameters. The numbers adjacent to the nodes are approximate likelihood-ratio test results. Hydra rhodopsins serve as outgroup. Green, red and blue clades are three major groups according to NJ tree. They are mixed in ML tree.
Supplemental figure 3: Bayesian tree for all metazoan rhodopsin genes. It is based on WAG model and 4 Gamma parameters. The numbers adjacent to the nodes are posterior probability values. Hydra rhodopsins serve as outgroup. Green, red and blue clades are three major groups according to NJ tree. They are mixed in Bayesian tree.
Supplemental figure 4: Unrooted Bayesian tree for all microbial rhodopsin genes. The numbers adjacent to the nodes are posterior probability values. The length of branch reflects evolutionary divergence. Green branch means archaea taxon. Red branch means eukaryote taxon. Blue branch means bacteria taxon.
Supplemental figure 5: Unrooted maximum-likelihood tree for all microbial rhodopsin genes. The numbers adjacent to the nodes are approximate likelihood-ratio test results. The length of branch reflects evolutionary divergence. Green branch means archaea taxon. Red branch means eukaryote taxon. Blue branch means bacteria taxon.
Supplemental figure 6: Unrooted neighbor-joining tree for all microbial rhodopsin genes. The numbers adjacent to tree nodes are bootstrap values. The length of branch reflects evolutionary divergence. Green branch means archaea taxon. Red branch means eukaryote taxon. Blue branch means bacteria taxon.

## Figures and Tables

**Table 1:** PDB accession numbers for two metazoan rhodopsins and five microbial rhodopsins.

PDB number	Protein name	Species	Classification
1U19	Rhodopsin	*Bos taurus* (bovine)	Eukaryota (Animalia)
2Z73	Rhodopsin	*Todarodes pacificus* (Japanese flying squid)	Eukaryota (Animalia)
1GU8	Sensory rhodopsin II	*Natronobacterium pharaonis*	Archaea (Halobacteria)
1JV6	Bacteriorhodopsin	Halobacterium salinarum	Archaea (Halobacteria)
1XIO	*Anabaena* sensory rhodopsin	*Nostoc* sp. pcc 7120	Bacteria (Cyanobacteria)
3A7K	Halorhodopsin	*Natronomonas pharaonis* dsm 2160	Archaea (Halobacteria)
3DDL	Xanthorhodopsin	Salinibacter ruber	Bacteria (Sphingobacteria)

**Table 2:** The number of rhodopsin genes in each metazoan species.

Common name	Phylum	Scientific name	Number of rhodopsins
Sponge	Porifera	*Amphimedon queenslandica*	0
*Hydra*	Cnidaria	*Hydra magnipapillata*	4
Nematode	Nematoda	*Caenorhabditis elegans*	0
Carolina anole	Chordata	*Anolis carolinensis*	15
Malaria mosquito	Arthropoda	*Anopheles gambiae*	9
Honey bee	Arthropoda	*Apis mellifera*	5
Bovine	Chordata	*Bos taurus*	6
Amphioxus	Chordata	*Branchiostoma floridae*	20
Dog	Chordata	*Canis lupus familiaris*	6
Sea squirt	Chordata	*Ciona intestinalis*	5
Zebra fish	Chordata	*Danio rerio*	35
Armadillo	Chordata	*Dasypus novemcinctus*	2
Fruit fly	Arthropoda	*Drosophila melanogaster*	6
Atlantic cod	Chordata	*Gadus morhua*	25
Chicken	Chordata	*Gallus gallus*	12
Human	Chordata	*Homo sapiens*	8
Coelacanth	Chordata	*Latimeria chalumnae*	11
Opossum	Chordata	*Monodelphis domestica*	8
Mouse	Chordata	*Mus musculus*	8
Brown bat	Chordata	*Myotis lucifugus*	6
Platypus	Chordata	*Ornithorhynchus anatinus*	4
Body louse	Arthropoda	*Pediculus humanus*	3
Lamprey	Chordata	*Petromyzon marinus*	3
Acorn worm	Hemichordata	*Saccoglossus kowalevskii*	1
Sea urchin	Echinodermata	*Strongylocentrotus purpuratus*	2
Dolphin	Chordata	*Tursiops truncatus*	5
Clawed frog	Chordata	*Xenopus tropicalis*	17

**Table 3:** Mutation distance between 24 metazoan rhodopsin's ancestral states and their evolutionarily closest microbial rhodopsin's ancestral state (Figure 3). Within test region, the average mutation distance between existent microbial and metazoan rhodopsins is 119 ± 5 mutations in the first and second codon positions.

	Observed mutation distance	Expected mutation distance (standard deviation)	The probability of observed mutation distance is caused by chance
Carolina anole	66	79.7 (±6.5)	0.0183 (<0.05)
Malaria mosquito	72	90.1 (±6.5)	0.0029 (<0.05)
Honey bee	79	84.9 (±6.6)	0.1814
Bovine	81	87.1 (±6.6)	0.1736
Amphioxus	75	92.9 (±6.5)	0.0031 (<0.05)
Dog	80	89.3 (±6.6)	0.0778
Sea squirt	68	84.5 (±6.6)	0.006 (<0.05)
Zebra fish	77	92.8 (±6.5)	0.0078 (<0.05)
Armadillo	75	82.4 (±6.6)	0.1292
Fruit fly	74	86.4 (±6.6)	0.0294 (<0.05)
Atlantic cod	86	92.4 (±6.5)	0.1611
Chicken	63	80.1 (±6.5)	0.0045 (<0.05)
Human	69	81.8 (±6.5)	0.0256 (<0.05)
Coelacanth	69	78.7 (±6.5)	0.0681
Opossum	67	82.6 (±6.6)	0.0084 (<0.05)
Mouse	69	73.5 (±6.5)	0.2451
Brown bat	74	74.8 (±6.5)	0.4522
Platypus	80	92.3 (±6.5)	0.0294 (<0.05)
Body louse	70	86.8 (±6.6)	0.0052 (<0.05)
Lamprey	68	78.4 (±6.5)	0.0548
Acorn worm	87	105.8 (±6.4)	0.0016 (<0.05)
Sea urchin	73	84.5 (±6.6)	0.0392 (<0.05)
Dolphin	69	79.6 (±6.5)	0.0516
Clawed frog	83	93 (±6.5)	0.063
