# Supplementary material for: The Evolutionary Relationship between Microbial Rhodopsins and Metazoan Rhodopsins
Source: ScientificWorldJournal. 2013 Feb 11;2013:435651. doi: 10.1155/2013/435651 (PMC3583139; doi:10.1155/2013/435651)

Supplemental table 1. Rhodopsin genes identified in microbial genomes.

NCBI accession No.	Protein name	Species	Tree name in this study
21218339	sensory opsin B	Chlamydomonas reinhardtii (Eukaryote)	Euk_Chl_s1
21218337	sensory opsin A	Chlamydomonas reinhardtii (Eukaryote)	Euk_Chl_s2
58397464	opsin	Cryptococcus neoformans var. neoformans (Eukaryote)	Euk_Cry_o1
NP_279315.1	bacteriorhodopsin	Halobacterium sp. NRC-1 (Archaea)	Arc_Hal_H4
YP_656804.1	bacteriorhodopsin	Haloquadratum walsbyi DSM 16790 (Archaea)	Arc_Hal_b1
YP_137573.1	bacteriorhodopsin	Haloarcula marismortui ATCC 43049 (Archaea)	Arc_Hal_b2
YP_001689404.1	bacteriorhodopsin	Halobacterium salinarum R1 (Archaea)	Arc_Hal_b3
YP_136594.1	bacteriorhodopsin	Haloarcula marismortui ATCC 43049 (Archaea)	Arc_Hal_b4
YP_656801.1	bacteriorhodopsin	Haloquadratum walsbyi DSM 16790 (Archaea)	Arc_Hal_b5
YP_004796777.1	halorhodopsin	Haloarcula hispanica ATCC 33960 (Archaea)	Arc_Hal_h1
YP_136278.1	halorhodopsin	Haloarcula marismortui ATCC 43049 (Archaea)	Arc_Hal_h2
YP_658762.1	halorhodopsin	Haloquadratum walsbyi DSM 16790 (Archaea)	Arc_Hal_h3
YP_003129368.1	rhodopsin	Halorhabdus utahensis DSM 12940 (Archaea)	Arc_Hal_m1
YP_135281.1	opsin	Haloarcula marismortui ATCC 43049 (Archaea)	Arc_Hal_o1
YP_002565246.1	rhodopsin	Halorubrum lacusprofundi ATCC 49239 (Archaea)	Arc_Hal_r1
YP_003176891.1	rhodopsin	Halomicrobium mukohataei DSM 12286 (Archaea)	Arc_Hal_r2
YP_002564761.1	rhodopsin	Halorubrum lacusprofundi ATCC 49239 (Archaea)	Arc_Hal_r3
YP_004596944.1	rhodopsin	Halopiger xanaduensis SH-6 (Archaea)	Arc_Hal_r4
YP_003178493.1	rhodopsin	Halomicrobium	Arc_Hal_r5

		mukohataei DSM 12286 (Archaea)	
YP_003129235.1	rhodopsin	Halorhabdus utahensis DSM 12940 (Archaea)	Arc_Hal_r6
YP_003177111.1	rhodopsin	Halomicrobium mukohataei DSM 12286 (Archaea)	Arc_Hal_r7
YP_001689625.1	sensory rhodopsin II	Halobacterium salinarum R1 (Archaea)	Arc_Hal_s1
YP_004795387.1	sensory rhodopsin 1	Haloarcula hispanica ATCC 33960 (Archaea)	Arc_Hal_s2
YP_001689548.1	sensory rhodopsin I	Halobacterium salinarum R1 (Archaea)	Arc_Hal_t1
YP_325981.1	halorhodopsin	Natronomonas pharaonis DSM 2160 (Archaea)	Arc_Nat_h1
YP_003480703.1	rhodopsin	Natrialba magadii ATCC 43099 (Archaea)	Arc_Nat_r1
YP_331142.1	sensory rhodopsin II	Natronomonas pharaonis DSM 2160 (Archaea)	Arc_Nat_s1
YP_266049.1	bacteriorhodopsin	Candidatus Pelagibacter ubique HTCC1062 (Bacteria)	Bac_Can_b1
YP_003552453.1	hypothetical protein	Candidatus Puniceispirillum marinum IMCC1322 (Bacteria)	Bac_Can_h1
YP_002484126.1	rhodopsin	Cyanothece sp. PCC 7425 (Bacteria)	Bac_Cya_r1
YP_002379171.1	rhodopsin	Cyanothece sp. PCC 7424 (Bacteria)	Bac_Cya_r2
YP_001813902.1	rhodopsin	Exiguobacterium sibiricum 255-15 (Bacteria)	Bac_Exi_r1
YP_002885111.1	rhodopsin	Exiguobacterium sp. AT1b (Bacteria)	Bac_Exi_r2
YP_003410608.1	rhodopsin	Geodermatophilus obscurus DSM 43160 (Bacteria)	Bac_Geo_r1
YP_004870549.1	bacteriorhodopsin	Glaciecola nitratireducens FR1064 (Bacteria)	Bac_Gla_b1
NP_923144.1	bacteriorhodopsin	Gloeobacter violaceus PCC 7421 (Bacteria)	Bac_Glo_b1
YP_004446506.1	rhodopsin	Haliscomenobacter hydrossis DSM 1100 (Bacteria)	Bac_Hal_r1

YP_004180611.1	rhodopsin	Isosphaera pallida ATCC 43644 (Bacteria)	Bac_Iso_r1
YP_001361545.1	rhodopsin	Kineococcus radiotolerans SRS30216 (Bacteria)	Bac_Kin_r1
YP_004429763.1	rhodopsin	Krokinobacter sp. 4H-3-7-5 (Bacteria)	Bac_Kro_r1
YP_004431469.1	rhodopsin	Krokinobacter sp. 4H-3-7-5 (Bacteria)	Bac_Kro_r2
EFH82634.1	rhodopsin	Ktedonobacter racemifer DSM 44963 (Bacteria)	Bac_Kte_r1
YP_004054869.1	rhodopsin	Marivirga tractuosa DSM 4126 (Bacteria)	Bac_Mar_r1
EAV46596.1	hypothetical protein	Methylophilales bacterium HTCC2181 (Bacteria)	Bac_Met_h1
YP_001767533.1	rhodopsin	Methylobacterium sp. 4-46 (Bacteria)	Bac_Met_r1
NP_487205.1	bacteriorhodopsin	Nostoc sp. PCC 7120 (Bacteria)	Bac_Nos_b1
YP_003931488.1	archaerhodopsin-1	Pantoea vagans C9-1 (Bacteria)	Bac_Pan_h1
YP_001277280.1	rhodopsin	Roseiflexus sp. RS-1 (Bacteria)	Bac_Ros_r1
YP_644794.1	rhodopsin	Rubrobacter xylanophilus DSM 9941 (Bacteria)	Bac_Rub_r1
YP_004653826.1	rhodopsin	Runella slithyformis DSM 19594 (Bacteria)	Bac_Run_r1
EHI47868.1	bacteriorhodopsin	SAR116 cluster alpha proteobacterium HIMB100 (Bacteria)	Bac_SAR_B1
YP_003572867.1	halorhodopsin	Salinibacter ruber M8 (Bacteria)	Bac_Sal_H1
YP_003571571.1	xanthorhodopsin	Salinibacter ruber M8 (Bacteria)	Bac_Sal_X1
YP_003572671.1	sensory rhodopsin I	Salinibacter ruber M8 (Bacteria)	Bac_Sal_s1
YP_446609.1	sensory rhodopsin (SRI)	Salinibacter ruber DSM 13855 (Bacteria)	Bac_Sal_s2
YP_003391271.1	rhodopsin	Spirosoma linguale DSM 74 (Bacteria)	Bac_Spi_r1
YP_003386489.1	rhodopsin	Spirosoma linguale DSM 74 (Bacteria)	Bac_Spi_r2

YP_004537746.1	rhodopsin	Thioalkalimicrobium cyclicum ALM1 (Bacteria)	Bac_Thi_r1
YP_003705905.1	rhodopsin	Truepera radiovictrix DSM 17093 (Bacteria)	Bac_Tru_r1
YP_003706581.1	rhodopsin	Truepera radiovictrix DSM 17093 (Bacteria)	Bac_Tru_r2
YP_003706549.1	rhodopsin	Truepera radiovictrix DSM 17093 (Bacteria)	Bac_Tru_r3
YP_001445352.1	hypothetical protein	Vibrio harveyi ATCC BAA-1116 (Bacteria)	Bac_Vib_h1

Supplemental table 2. Microbial rhodopsin gene divergently related to metazoan rhodopsin's ancestral states and how many metazoan rhodopsin's ancestral states it is related to. Here 95% quantile = 15.25.

Tree name of microbial rhodopsin divergently related to metazoan rhodopsin's ancestral states	How many metazoan rhodopsin's ancestral states it is related to (out of 24)
Bac_Exi_r2	2
Bac_Pan_h1	4
Bac_Rub_r1	12
Arc_Hal_h2	11
Bac_SAR_B1	2
Bac_Kin_r1	10
Bac_Cya_r1	7
Bac_Thi_r1	1
Arc_Hal_r3	12
Arc_Hal_r5	4
Arc_Hal_m1	2
Bac_Ros_r1	5
Bac_Tru_r1	7
Bac_Spi_r2	4
Arc_Hal_s1	1
Arc_Hal_s2	2
Arc_Hal_b5	1
Bac_Mar_r1	1
Bac_Can_b1	4
Arc_Hal_r7	1
Bac_Kro_r2	2
Bac_Geo_r1	1
Bac_Can_h1	4
Bac_Sal_s1	5
Arc_Hal_t1	2

Bac_Tru_r2	12
Arc_Hal_r1	15
Arc_Hal_b3	5
Bac_Exi_r1	6
Euk_Chl_s1	7
Bac_Iso_r1	1
Bac_Glo_b1	3
Bac_Nos_b1	3
Bac_Sal_X1	6
Bac_Met_r1	3
Bac_Kro_r1	4
Arc_Hal_b4	1
Arc_Nat_h1	17 (> 95% quantile)
Arc_Hal_b1	1
Arc_Hal_r4	3
Arc_Nat_r1	16 (> 95% quantile)
Bac_Hal_r1	1
Arc_Hal_h1	10
Bac_Spi_r1	1
Bac_Tru_r3	2
Arc_Hal_r2	12
Bac_Sal_H1	10
Arc_Hal_o1	3
Arc_Hal_H4	11
Bac_Met_h1	9
Euk_Cry_o1	5
Arc_Nat_s1	1
Bac_Gla_b1	8
Euk_Chl_s2	6
Bac_Run_r1	3
Arc_Hal_h3	16 (> 95% quantile)

Supplemental figure legends

Supplemental figure 1. Neighbor-joining tree for all metazoan rhodopsin genes. It is based on JTT model and 4 Gamma parameters. The numbers adjacent to tree nodes are bootstrap values. Hydra rhodopsins serve as outgroup. Green, red and blue clades are three major groups in this tree.

Supplemental figure 2. Maximum-likelihood tree for all metazoan rhodopsin genes. It is based on WAG model and 4 Gamma parameters. The numbers adjacent to the nodes are approximate likelihood-ratio test results. Hydra rhodopsins serve as outgroup. Green, red and blue clades are three major groups according to NJ tree. They are mixed in ML tree.

Supplemental figure 3. Bayesian tree for all metazoan rhodopsin genes. It is based on WAG model and 4 Gamma parameters. The numbers adjacent to the nodes are posterior probability values. Hydra rhodopsins serve as outgroup. Green, red and blue clades are three major groups according to NJ tree. They are mixed in Bayesian tree.

Supplemental figure 4. Unrooted Bayesian tree for all microbial rhodopsin genes. The numbers adjacent to the nodes are posterior probability values. The length of branch reflects evolutionary divergence. Green branch means archaea taxon. Red branch means eukaryote taxon. Blue branch means bacteria taxon.

Supplemental figure 5. Unrooted maximum-likelihood tree for all microbial rhodopsin genes. The numbers adjacent to the nodes are approximate likelihood-ratio test results. The length of branch reflects evolutionary divergence. Green branch means archaea taxon. Red branch means eukaryote taxon. Blue branch means bacteria taxon.

Supplemental figure 6. Unrooted neighbor-joining tree for all microbial rhodopsin genes. The numbers adjacent to tree nodes are bootstrap values. The length of branch reflects evolutionary divergence. Green branch means archaea taxon. Red branch means eukaryote taxon. Blue branch means bacteria taxon.

Supplemental figure 1.

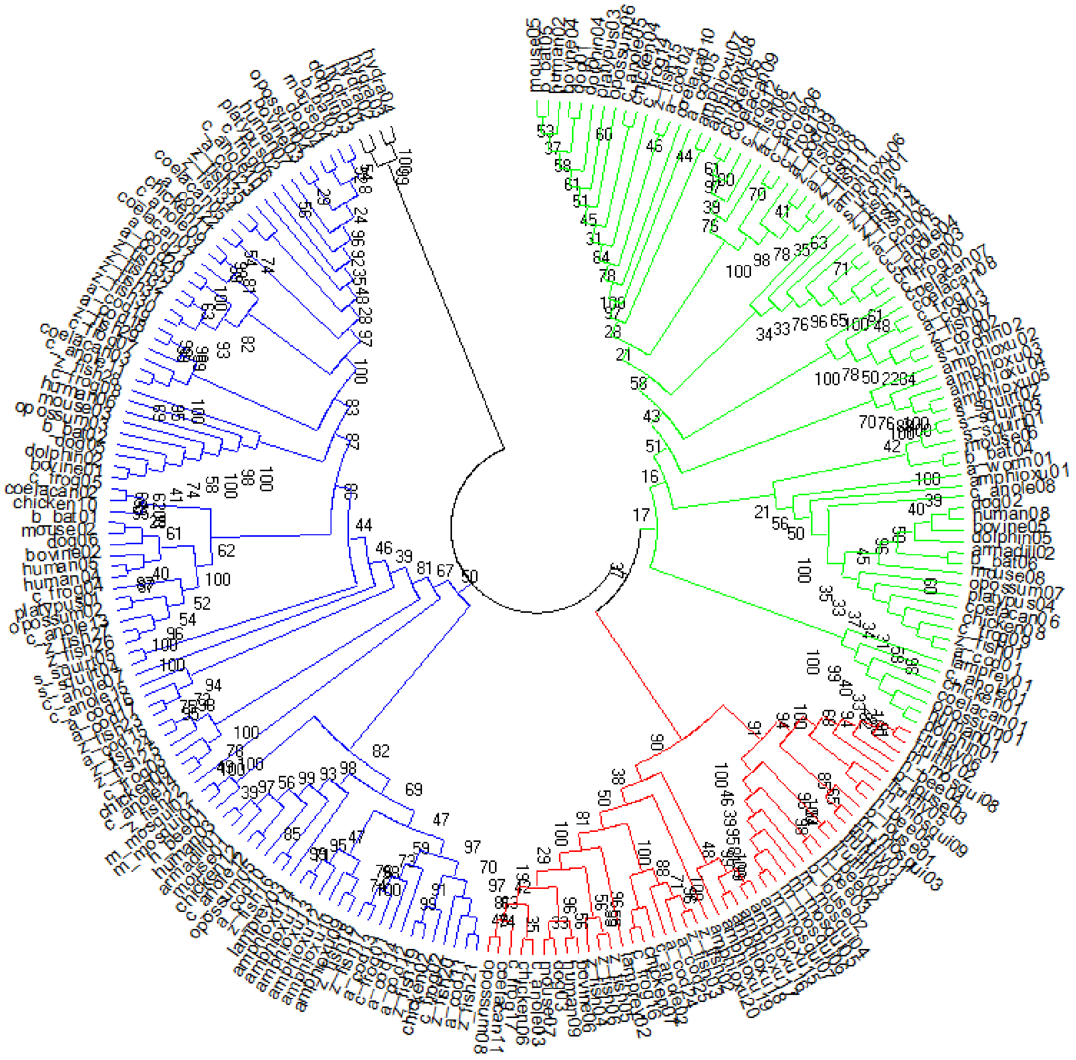

Supplemental figure 2.

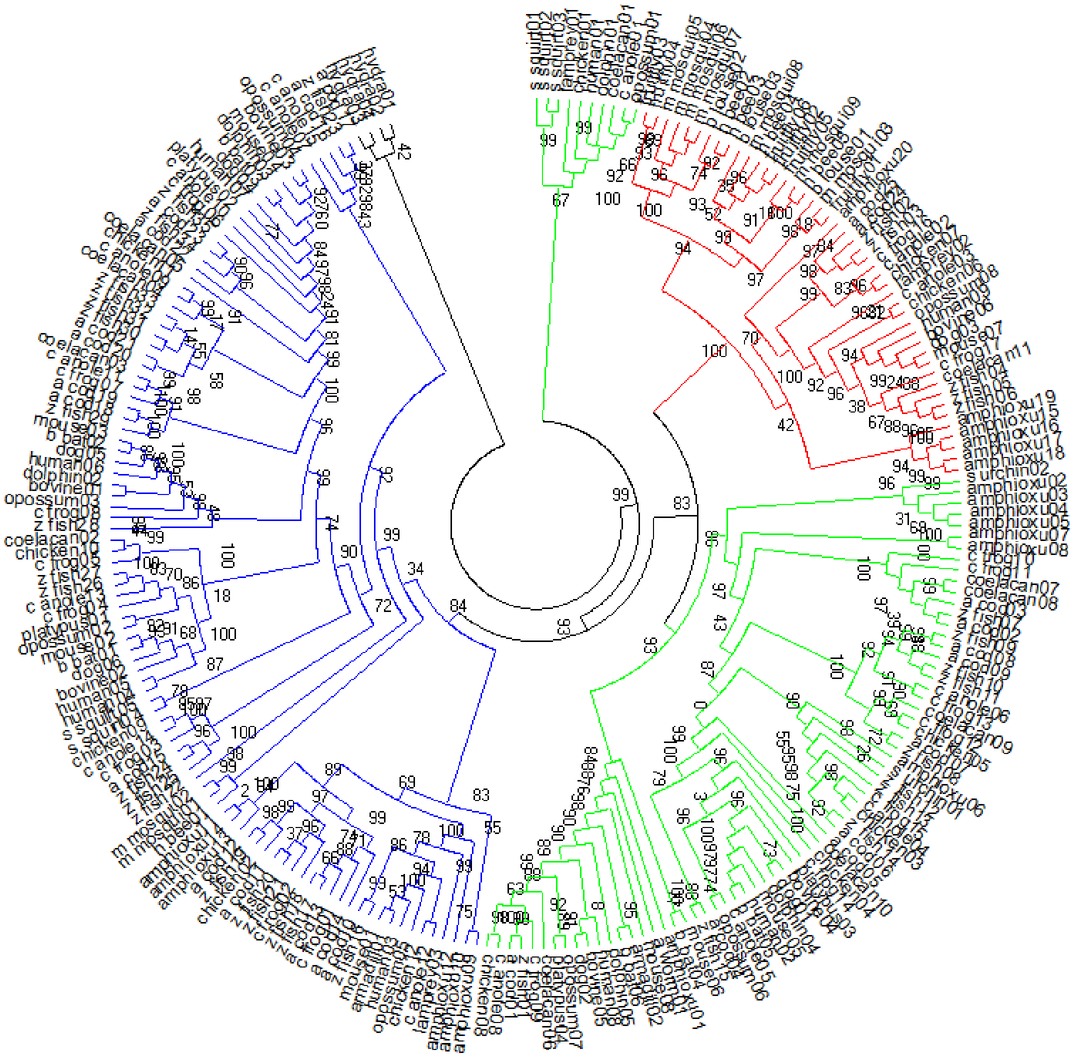

Supplemental figure 3.

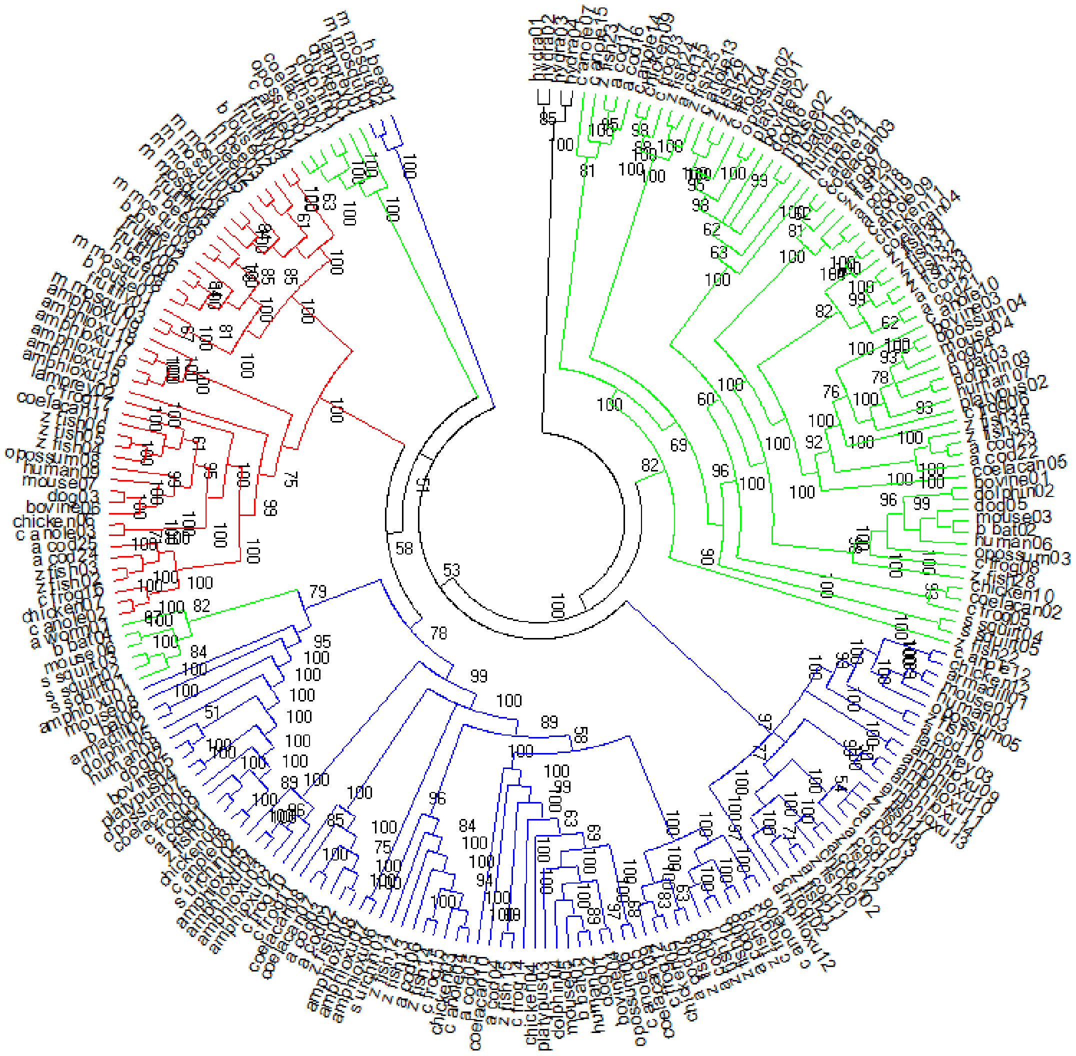

Supplemental figure 4.

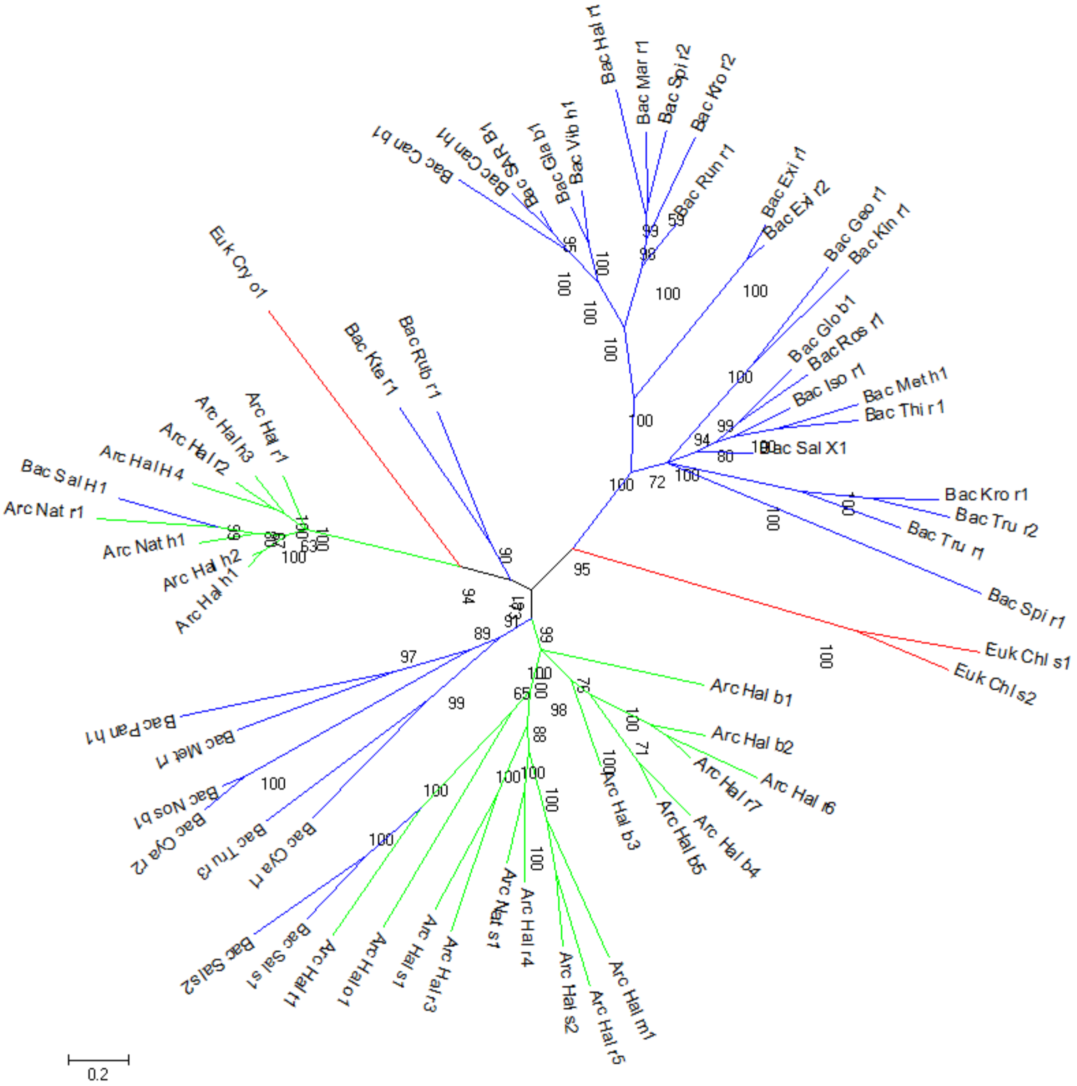

Supplemental figure 5.

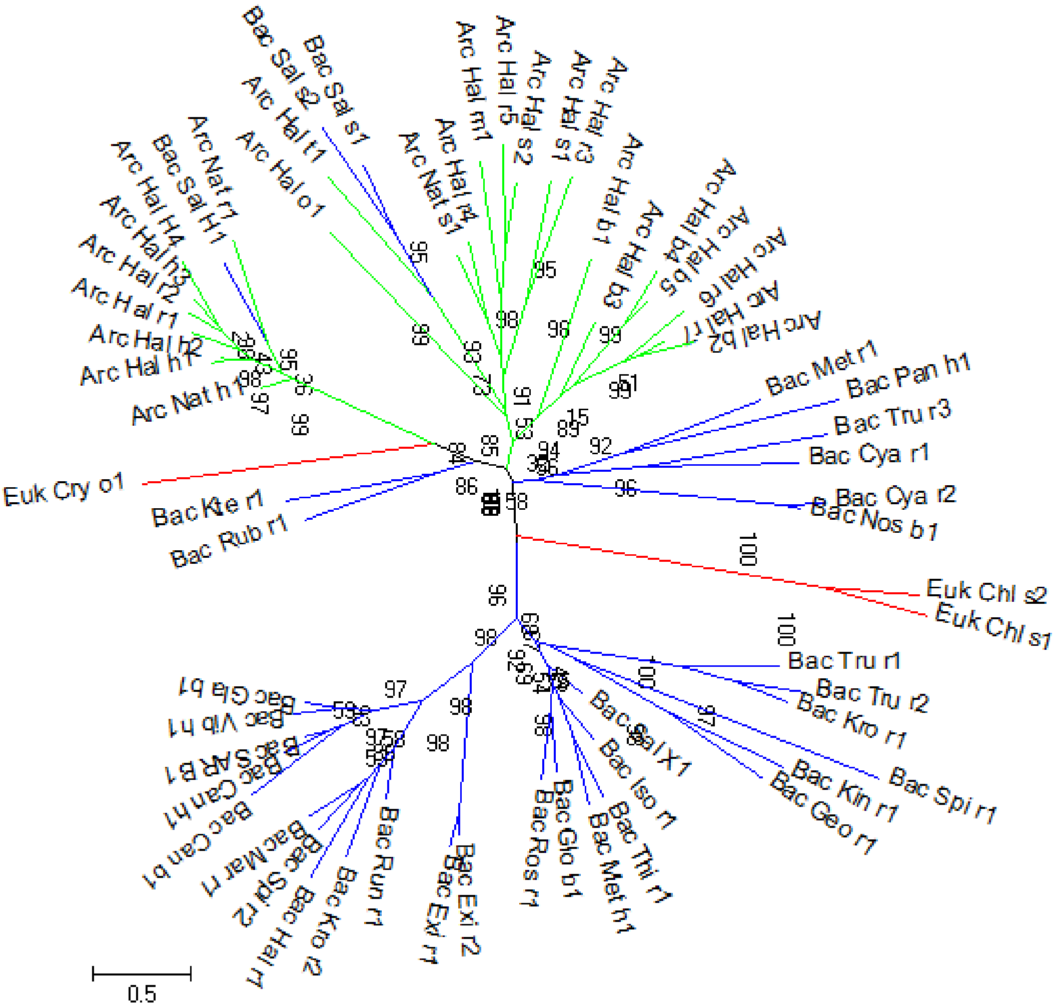

Supplemental figure 6.

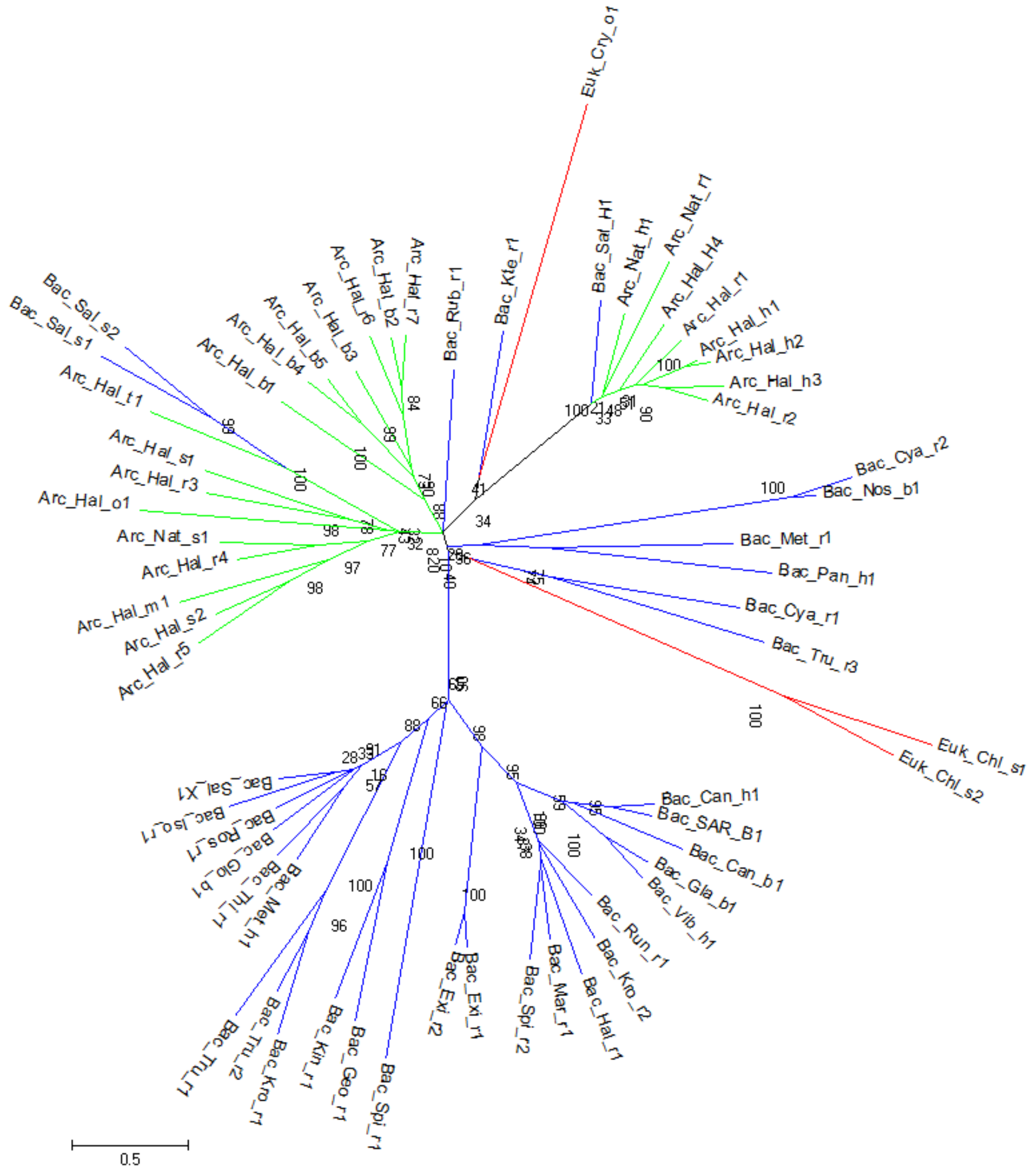

Supplement: Supplementary file 1 — Supplemental table 1: Rhodopsin genes identified in microbial genomes. Supplemental table 2: Microbial rhodopsin gene divergently related to metazoan rhodopsin's ancestral states and how many metazoan rhodopsin's ancestral states it is related to. Here 95% quantile = 15.25. Supplemental figure 1: Neighbor-joining tree for all metazoan rhodopsin genes. It is based on JTT model and 4 Gamma parameters. The numbers adjacent to tree nodes are bootstrap values. Hydra rhodopsins serve as outgroup. Green, red and blue clades are three major groups in this tree. Supplemental figure 2: Maximum-likelihood tree for all metazoan rhodopsin genes. It is based on WAG model and 4 Gamma parameters. The numbers adjacent to the nodes are approximate likelihood-ratio test results. Hydra rhodopsins serve as outgroup. Green, red and blue clades are three major groups according to NJ tree. They are mixed in ML tree. Supplemental figure 3: Bayesian tree for all metazoan rhodopsin genes. It is based on WAG model and 4 Gamma parameters. The numbers adjacent to the nodes are posterior probability values. Hydra rhodopsins serve as outgroup. Green, red and blue clades are three major groups according to NJ tree. They are mixed in Bayesian tree. Supplemental figure 4: Unrooted Bayesian tree for all microbial rhodopsin genes. The numbers adjacent to the nodes are posterior probability values. The length of branch reflects evolutionary divergence. Green branch means archaea taxon. Red branch means eukaryote taxon. Blue branch means bacteria taxon. Supplemental figure 5: Unrooted maximum-likelihood tree for all microbial rhodopsin genes. The numbers adjacent to the nodes are approximate likelihood-ratio test results. The length of branch reflects evolutionary divergence. Green branch means archaea taxon. Red branch means eukaryote taxon. Blue branch means bacteria taxon. Supplemental figure 6: Unrooted neighbor-joining tree for all microbial rhodopsin genes. The numbers adjacent to tree nodes are bo [file 435651.f1.pdf]
